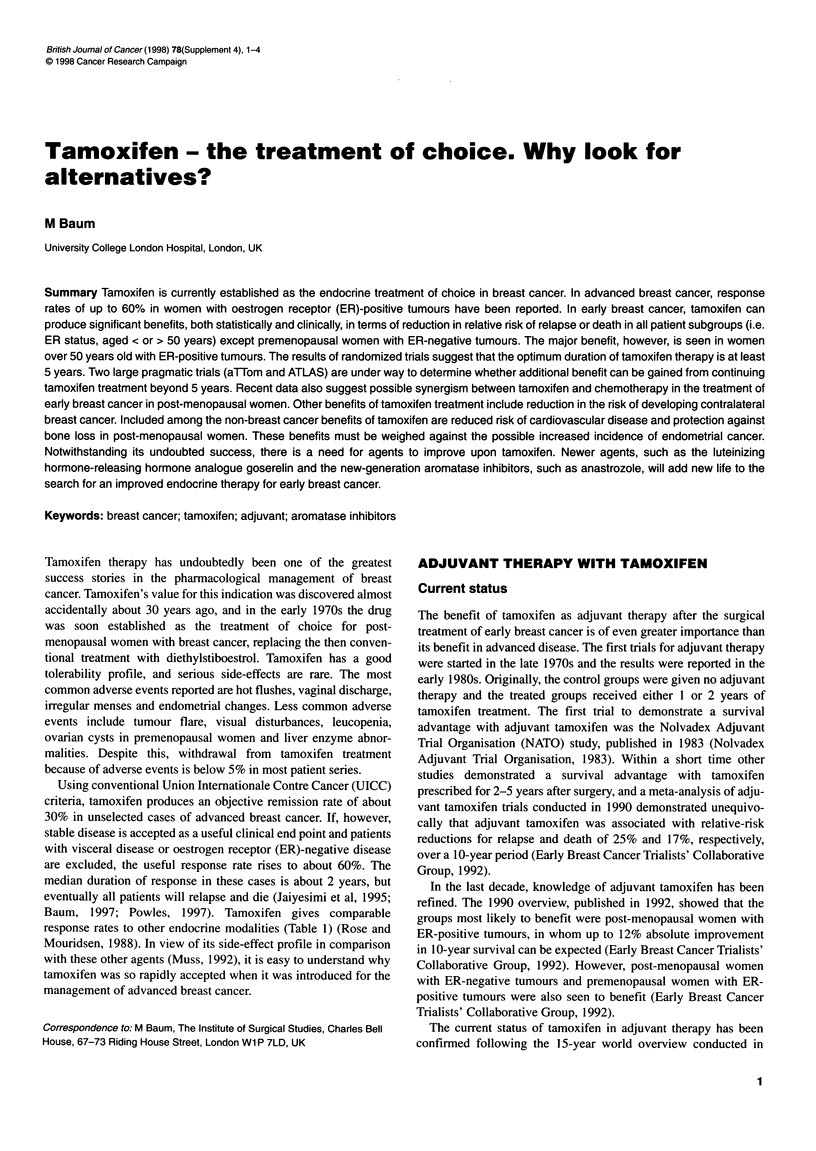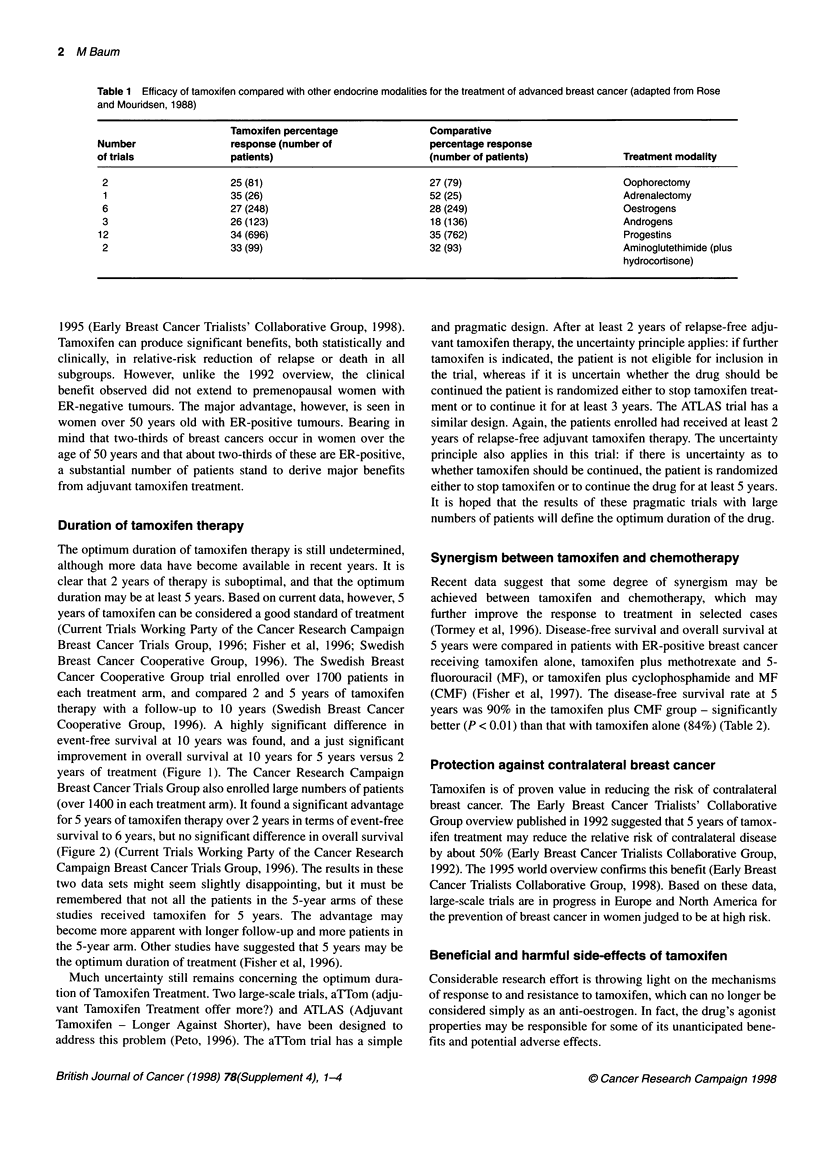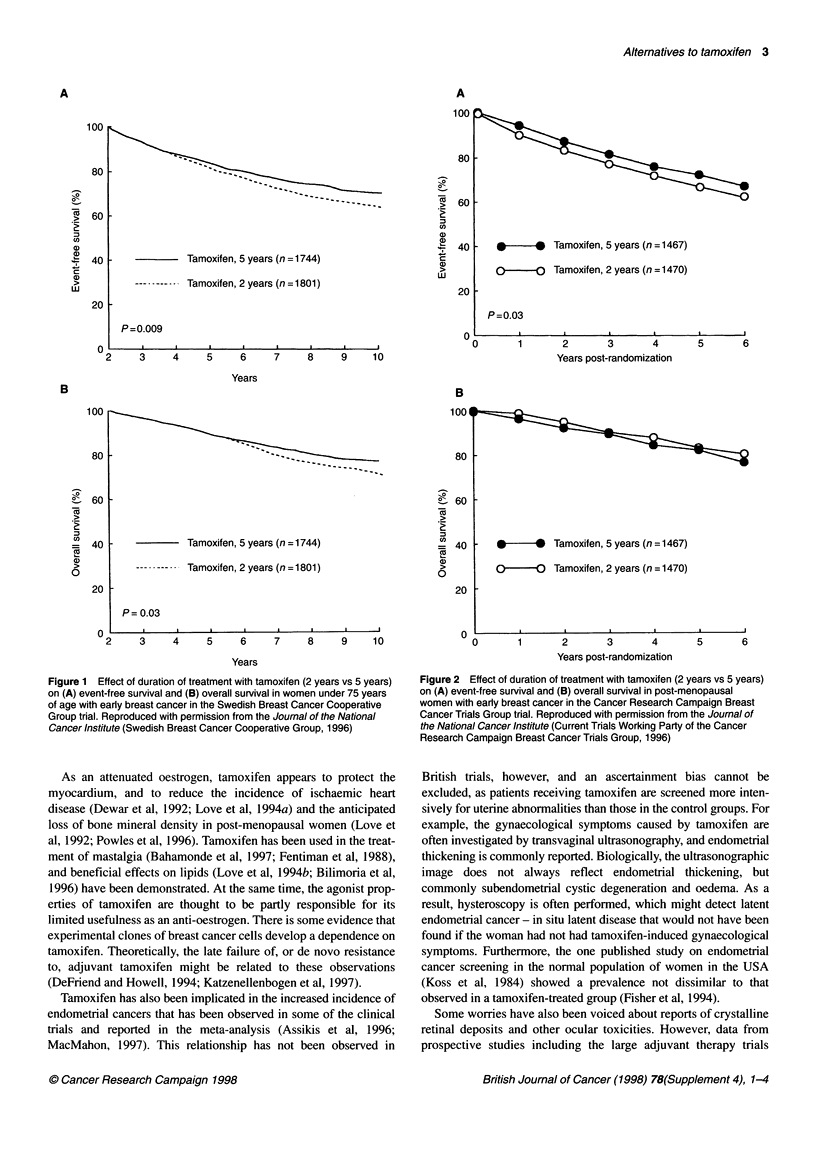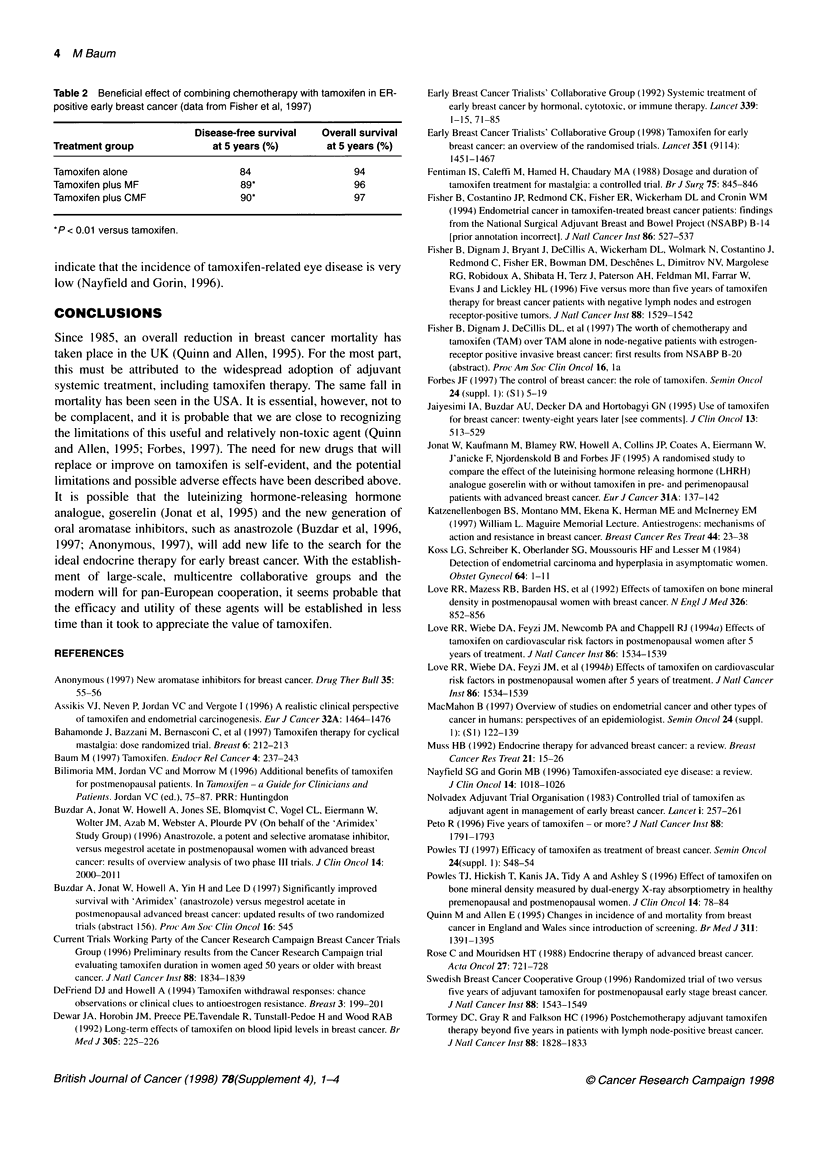# Tamoxifen--the treatment of choice. Why look for alternatives?

**DOI:** 10.1038/bjc.1998.753

**Published:** 1998-09

**Authors:** M. Baum

**Affiliations:** University College London Hospital, UK.

## Abstract

Tamoxifen is currently established as the endocrine treatment of choice in breast cancer. In advanced breast cancer, response rates of up to 60% in women with oestrogen receptor (ER)-positive tumours have been reported. In early breast cancer, tamoxifen can produce significant benefits, both statistically and clinically, in terms of reduction in relative risk of relapse or death in all patient subgroups (i.e. ER status, aged < or > 50 years) except premenopausal women with ER-negative tumours. The major benefit, however, is seen in women over 50 years old with ER-positive tumours. The results of randomized trials suggest that the optimum duration of tamoxifen therapy is at least 5 years. Two large pragmatic trials (aTTom and ATLAS) are under way to determine whether additional benefit can be gained from continuing tamoxifen treatment beyond 5 years. Recent data also suggest possible synergism between tamoxifen and chemotherapy in the treatment of early breast cancer in post-menopausal women. Other benefits of tamoxifen treatment include reduction in the risk of developing contralateral breast cancer. Included among the non-breast cancer benefits of tamoxifen are reduced risk of cardiovascular disease and protection against bone loss in post-menopausal women. These benefits must be weighed against the possible increased incidence of endometrial cancer. Notwithstanding its undoubted success, there is a need for agents to improve upon tamoxifen. Newer agents, such as the luteinizing hormone-releasing hormone analogue goserelin and the new-generation aromatase inhibitors, such as anastrozole, will add new life to the search for an improved endocrine therapy for early breast cancer.


British Journal of Cancer (1998) 78(Supplement 4), 1–4
© 1998 Cancer Research Campaign

### Tamoxifen – the treatment of choice. Why look for alternatives?

**M Baum**

University College London Hospital, London, UK

**Summary** Tamoxifen is currently established as the endocrine treatment of choice in breast cancer. In advanced breast cancer, response rates of up to 60% in women with oestrogen receptor (ER)-positive tumours have been reported. In early breast cancer, tamoxifen can produce significant benefits, both statistically and clinically, in terms of reduction in relative risk of relapse or death in all patient subgroups (i.e. ER status, aged < or > 50 years) except premenopausal women with ER-negative tumours. The major benefit, however, is seen in women over 50 years old with ER-positive tumours. The results of randomized trials suggest that the optimum duration of tamoxifen therapy is at least 5 years. Two large pragmatic trials (aTTom and ATLAS) are under way to determine whether additional benefit can be gained from continuing tamoxifen treatment beyond 5 years. Recent data also suggest possible synergism between tamoxifen and chemotherapy in the treatment of early breast cancer in post-menopausal women. Other benefits of tamoxifen treatment include reduction in the risk of developing contralateral breast cancer. Included among the non-breast cancer benefits of tamoxifen are reduced risk of cardiovascular disease and protection against bone loss in post-menopausal women. These benefits must be weighed against the possible increased incidence of endometrial cancer. Notwithstanding its undoubted success, there is a need for agents to improve upon tamoxifen. Newer agents, such as the luteinizing hormone-releasing hormone analogue goserelin and the new-generation aromatase inhibitors, such as anastrozole, will add new life to the search for an improved endocrine therapy for early breast cancer.

**Keywords:** breast cancer; tamoxifen; adjuvant; aromatase inhibitors

Tamoxifen therapy has undoubtedly been one of the greatest success stories in the pharmacological management of breast cancer. Tamoxifen's value for this indication was discovered almost accidentally about 30 years ago, and in the early 1970s the drug was soon established as the treatment of choice for post-menopausal women with breast cancer, replacing the then conventional treatment with diethylstiboestrol. Tamoxifen has a good tolerability profile, and serious side-effects are rare. The most common adverse events reported are hot flushes, vaginal discharge, irregular menses and endometrial changes. Less common adverse events include tumour flare, visual disturbances, leucopenia, ovarian cysts in premenopausal women and liver enzyme abnormalities. Despite this, withdrawal from tamoxifen treatment because of adverse events is below 5% in most patient series.

Using conventional Union Internationale Contre Cancer (UICC) criteria, tamoxifen produces an objective remission rate of about 30% in unselected cases of advanced breast cancer. If, however, stable disease is accepted as a useful clinical end point and patients with visceral disease or oestrogen receptor (ER)-negative disease are excluded, the useful response rate rises to about 60%. The median duration of response in these cases is about 2 years, but eventually all patients will relapse and die (Jaiyesimi et al, 1995; Baum, 1997; Powles, 1997). Tamoxifen gives comparable response rates to other endocrine modalities (Table 1) (Rose and Mouridsen, 1988). In view of its side-effect profile in comparison with these other agents (Muss, 1992), it is easy to understand why tamoxifen was so rapidly accepted when it was introduced for the management of advanced breast cancer.

*Correspondence to:* M Baum, The Institute of Surgical Studies, Charles Bell House, 67–73 Riding House Street, London W1P 7LD, UK

### ADJUVANT THERAPY WITH TAMOXIFEN

### Current status

The benefit of tamoxifen as adjuvant therapy after the surgical treatment of early breast cancer is of even greater importance than its benefit in advanced disease. The first trials for adjuvant therapy were started in the late 1970s and the results were reported in the early 1980s. Originally, the control groups were given no adjuvant therapy and the treated groups received either 1 or 2 years of tamoxifen treatment. The first trial to demonstrate a survival advantage with adjuvant tamoxifen was the Nolvadex Adjuvant Trial Organisation (NATO) study, published in 1983 (Nolvadex Adjuvant Trial Organisation, 1983). Within a short time other studies demonstrated a survival advantage with tamoxifen prescribed for 2–5 years after surgery, and a meta-analysis of adjuvant tamoxifen trials conducted in 1990 demonstrated unequivocally that adjuvant tamoxifen was associated with relative-risk reductions for relapse and death of 25% and 17%, respectively, over a 10-year period (Early Breast Cancer Trialists' Collaborative Group, 1992).

In the last decade, knowledge of adjuvant tamoxifen has been refined. The 1990 overview, published in 1992, showed that the groups most likely to benefit were post-menopausal women with ER-positive tumours, in whom up to 12% absolute improvement in 10-year survival can be expected (Early Breast Cancer Trialists' Collaborative Group, 1992). However, post-menopausal women with ER-negative tumours and premenopausal women with ER-positive tumours were also seen to benefit (Early Breast Cancer Trialists' Collaborative Group, 1992).

The current status of tamoxifen in adjuvant therapy has been confirmed following the 15-year world overview conducted in

**Table 1** Efficacy of tamoxifen compared with other endocrine modalities for the treatment of advanced breast cancer (adapted from Rose and Mouridsen, 1988)

| Number of trials | Tamoxifen percentage response (number of patients) | Comparative percentage response (number of patients) | Treatment modality |
|---|---|---|---|
| 2 | 25 (81) | 27 (79) | Oophorectomy |
| 1 | 35 (26) | 52 (25) | Adrenalectomy |
| 6 | 27 (248) | 28 (249) | Oestrogens |
| 3 | 26 (123) | 18 (136) | Androgens |
| 12 | 34 (696) | 35 (762) | Progestins |
| 2 | 33 (99) | 32 (93) | Aminoglutethimide (plus hydrocortisone) |

1995 (Early Breast Cancer Trialists' Collaborative Group, 1998). Tamoxifen can produce significant benefits, both statistically and clinically, in relative-risk reduction of relapse or death in all subgroups. However, unlike the 1992 overview, the clinical benefit observed did not extend to premenopausal women with ER-negative tumours. The major advantage, however, is seen in women over 50 years old with ER-positive tumours. Bearing in mind that two-thirds of breast cancers occur in women over the age of 50 years and that about two-thirds of these are ER-positive, a substantial number of patients stand to derive major benefits from adjuvant tamoxifen treatment.

### Duration of tamoxifen therapy

The optimum duration of tamoxifen therapy is still undetermined, although more data have become available in recent years. It is clear that 2 years of therapy is suboptimal, and that the optimum duration may be at least 5 years. Based on current data, however, 5 years of tamoxifen can be considered a good standard of treatment (Current Trials Working Party of the Cancer Research Campaign Breast Cancer Trials Group, 1996; Fisher et al, 1996; Swedish Breast Cancer Cooperative Group, 1996). The Swedish Breast Cancer Cooperative Group trial enrolled over 1700 patients in each treatment arm, and compared 2 and 5 years of tamoxifen therapy with a follow-up to 10 years (Swedish Breast Cancer Cooperative Group, 1996). A highly significant difference in event-free survival at 10 years was found, and a just significant improvement in overall survival at 10 years for 5 years versus 2 years of treatment (Figure 1). The Cancer Research Campaign Breast Cancer Trials Group also enrolled large numbers of patients (over 1400 in each treatment arm). It found a significant advantage for 5 years of tamoxifen therapy over 2 years in terms of event-free survival to 6 years, but no significant difference in overall survival (Figure 2) (Current Trials Working Party of the Cancer Research Campaign Breast Cancer Trials Group, 1996). The results in these two data sets might seem slightly disappointing, but it must be remembered that not all the patients in the 5-year arms of these studies received tamoxifen for 5 years. The advantage may become more apparent with longer follow-up and more patients in the 5-year arm. Other studies have suggested that 5 years may be the optimum duration of treatment (Fisher et al, 1996).

Much uncertainty still remains concerning the optimum duration of Tamoxifen Treatment. Two large-scale trials, aTTom (adjuvant Tamoxifen Treatment offer more?) and ATLAS (Adjuvant Tamoxifen – Longer Against Shorter), have been designed to address this problem (Peto, 1996). The aTTom trial has a simple and pragmatic design. After at least 2 years of relapse-free adjuvant tamoxifen therapy, the uncertainty principle applies: if further tamoxifen is indicated, the patient is not eligible for inclusion in the trial, whereas if it is uncertain whether the drug should be continued the patient is randomized either to stop tamoxifen treatment or to continue it for at least 3 years. The ATLAS trial has a similar design. Again, the patients enrolled had received at least 2 years of relapse-free adjuvant tamoxifen therapy. The uncertainty principle also applies in this trial: if there is uncertainty as to whether tamoxifen should be continued, the patient is randomized either to stop tamoxifen or to continue the drug for at least 5 years. It is hoped that the results of these pragmatic trials with large numbers of patients will define the optimum duration of the drug.

### Synergism between tamoxifen and chemotherapy

Recent data suggest that some degree of synergism may be achieved between tamoxifen and chemotherapy, which may further improve the response to treatment in selected cases (Tormey et al, 1996). Disease-free survival and overall survival at 5 years were compared in patients with ER-positive breast cancer receiving tamoxifen alone, tamoxifen plus methotrexate and 5-fluorouracil (MF), or tamoxifen plus cyclophosphamide and MF (CMF) (Fisher et al, 1997). The disease-free survival rate at 5 years was 90% in the tamoxifen plus CMF group – significantly better ($P < 0.01$) than that with tamoxifen alone (84%) (Table 2).

### Protection against contralateral breast cancer

Tamoxifen is of proven value in reducing the risk of contralateral breast cancer. The Early Breast Cancer Trialists' Collaborative Group overview published in 1992 suggested that 5 years of tamoxifen treatment may reduce the relative risk of contralateral disease by about 50% (Early Breast Cancer Trialists Collaborative Group, 1992). The 1995 world overview confirms this benefit (Early Breast Cancer Trialists Collaborative Group, 1998). Based on these data, large-scale trials are in progress in Europe and North America for the prevention of breast cancer in women judged to be at high risk.

### Beneficial and harmful side-effects of tamoxifen

Considerable research effort is throwing light on the mechanisms of response to and resistance to tamoxifen, which can no longer be considered simply as an anti-oestrogen. In fact, the drug's agonist properties may be responsible for some of its unanticipated benefits and potential adverse effects.

 © Cancer Research Campaign 1998

**Figure 1** Effect of duration of treatment with tamoxifen (2 years vs 5 years) on (**A**) event-free survival and (**B**) overall survival in women under 75 years of age with early breast cancer in the Swedish Breast Cancer Cooperative Group trial. Reproduced with permission from the *Journal of the National Cancer Institute* (Swedish Breast Cancer Cooperative Group, 1996)

**Figure 2** Effect of duration of treatment with tamoxifen (2 years vs 5 years) on (**A**) event-free survival and (**B**) overall survival in post-menopausal women with early breast cancer in the Cancer Research Campaign Breast Cancer Trials Group trial. Reproduced with permission from the *Journal of the National Cancer Institute* (Current Trials Working Party of the Cancer Research Campaign Breast Cancer Trials Group, 1996)

As an attenuated oestrogen, tamoxifen appears to protect the myocardium, and to reduce the incidence of ischaemic heart disease (Dewar et al, 1992; Love et al, 1994*a*) and the anticipated loss of bone mineral density in post-menopausal women (Love et al, 1992; Powles et al, 1996). Tamoxifen has been used in the treatment of mastalgia (Bahamonde et al, 1997; Fentiman et al, 1988), and beneficial effects on lipids (Love et al, 1994*b*; Bilimoria et al, 1996) have been demonstrated. At the same time, the agonist properties of tamoxifen are thought to be partly responsible for its limited usefulness as an anti-oestrogen. There is some evidence that experimental clones of breast cancer cells develop a dependence on tamoxifen. Theoretically, the late failure of, or de novo resistance to, adjuvant tamoxifen might be related to these observations (DeFriend and Howell, 1994; Katzenellenbogen et al, 1997).

Tamoxifen has also been implicated in the increased incidence of endometrial cancers that has been observed in some of the clinical trials and reported in the meta-analysis (Assikis et al, 1996; MacMahon, 1997). This relationship has not been observed in British trials, however, and an ascertainment bias cannot be excluded, as patients receiving tamoxifen are screened more intensively for uterine abnormalities than those in the control groups. For example, the gynaecological symptoms caused by tamoxifen are often investigated by transvaginal ultrasonography, and endometrial thickening is commonly reported. Biologically, the ultrasonographic image does not always reflect endometrial thickening, but commonly subendometrial cystic degeneration and oedema. As a result, hysteroscopy is often performed, which might detect latent endometrial cancer – in situ latent disease that would not have been found if the woman had not had tamoxifen-induced gynaecological symptoms. Furthermore, the one published study on endometrial cancer screening in the normal population of women in the USA (Koss et al, 1984) showed a prevalence not dissimilar to that observed in a tamoxifen-treated group (Fisher et al, 1994).

Some worries have also been voiced about reports of crystalline retinal deposits and other ocular toxicities. However, data from prospective studies including the large adjuvant therapy trials

© Cancer Research Campaign 1998

**Table 2** Beneficial effect of combining chemotherapy with tamoxifen in ER-positive early breast cancer (data from Fisher et al, 1997)

| Treatment group | Disease-free survival at 5 years (%) | Overall survival at 5 years (%) |
|---|---|---|
| Tamoxifen alone | 84 | 94 |
| Tamoxifen plus MF | 89* | 96 |
| Tamoxifen plus CMF | 90* | 97 |

*$P < 0.01$ versus tamoxifen.

indicate that the incidence of tamoxifen-related eye disease is very low (Nayfield and Gorin, 1996).

### CONCLUSIONS

Since 1985, an overall reduction in breast cancer mortality has taken place in the UK (Quinn and Allen, 1995). For the most part, this must be attributed to the widespread adoption of adjuvant systemic treatment, including tamoxifen therapy. The same fall in mortality has been seen in the USA. It is essential, however, not to be complacent, and it is probable that we are close to recognizing the limitations of this useful and relatively non-toxic agent (Quinn and Allen, 1995; Forbes, 1997). The need for new drugs that will replace or improve on tamoxifen is self-evident, and the potential limitations and possible adverse effects have been described above. It is possible that the luteinizing hormone-releasing hormone analogue, goserelin (Jonat et al, 1995) and the new generation of oral aromatase inhibitors, such as anastrozole (Buzdar et al, 1996, 1997; Anonymous, 1997), will add new life to the search for the ideal endocrine therapy for early breast cancer. With the establishment of large-scale, multicentre collaborative groups and the modern will for pan-European cooperation, it seems probable that the efficacy and utility of these agents will be established in less time than it took to appreciate the value of tamoxifen.

### REFERENCES

Anonymous (1997) New aromatase inhibitors for breast cancer. *Drug Ther Bull* **35**: 55–56

Assikis VJ, Neven P, Jordan VC and Vergote I (1996) A realistic clinical perspective of tamoxifen and endometrial carcinogenesis. *Eur J Cancer* **32A**: 1464–1476

Bahamonde J, Bazzani M, Bernasconi C, et al (1997) Tamoxifen therapy for cyclical mastalgia: dose randomized trial. *Breast* **6**: 212–213

Baum M (1997) Tamoxifen. *Endocr Rel Cancer* **4**: 237–243

Bilimoria MM, Jordan VC and Morrow M (1996) Additional benefits of tamoxifen for postmenopausal patients. In *Tamoxifen – a Guide for Clinicians and Patients*. Jordan VC (ed.), 75–87. PRR: Huntingdon

Buzdar A, Jonat W, Howell A, Jones SE, Blomqvist C, Vogel CL, Eiermann W, Wolter JM, Azab M, Webster A, Plourde PV (On behalf of the 'Arimidex' Study Group) (1996) Anastrozole, a potent and selective aromatase inhibitor, versus megestrol acetate in postmenopausal women with advanced breast cancer: results of overview analysis of two phase III trials. *J Clin Oncol* **14**: 2000–2011

Buzdar A, Jonat W, Howell A, Yin H and Lee D (1997) Significantly improved survival with 'Arimidex' (anastrozole) versus megestrol acetate in postmenopausal advanced breast cancer: updated results of two randomized trials (abstract 156). *Proc Am Soc Clin Oncol* **16**: 545

Current Trials Working Party of the Cancer Research Campaign Breast Cancer Trials Group (1996) Preliminary results from the Cancer Research Campaign trial evaluating tamoxifen duration in women aged 50 years or older with breast cancer. *J Natl Cancer Inst* **88**: 1834–1839

DeFriend DJ and Howell A (1994) Tamoxifen withdrawal responses: chance observations or clinical clues to antioestrogen resistance. *Breast* **3**: 199–201

Dewar JA, Horobin JM, Preece PE,Tavendale R, Tunstall-Pedoe H and Wood RAB (1992) Long-term effects of tamoxifen on blood lipid levels in breast cancer. *Br Med J* **305**: 225–226

Early Breast Cancer Trialists' Collaborative Group (1992) Systemic treatment of early breast cancer by hormonal, cytotoxic, or immune therapy. *Lancet* **339**: 1–15, 71–85

Early Breast Cancer Trialists' Collaborative Group (1998) Tamoxifen for early breast cancer: an overview of the randomised trials. *Lancet* **351** (9114): 1451–1467

Fentiman IS, Caleffi M, Hamed H, Chaudary MA (1988) Dosage and duration of tamoxifen treatment for mastalgia: a controlled trial. *Br J Surg* **75**: 845–846

Fisher B, Costantino JP, Redmond CK, Fisher ER, Wickerham DL and Cronin WM (1994) Endometrial cancer in tamoxifen-treated breast cancer patients: findings from the National Surgical Adjuvant Breast and Bowel Project (NSABP) B-14 [prior annotation incorrect]. *J Natl Cancer Inst* **86**: 527–537

Fisher B, Dignam J, Bryant J, DeCillis A, Wickerham DL, Wolmark N, Costantino J, Redmond C, Fisher ER, Bowman DM, Deschênes L, Dimitrov NV, Margolese RG, Robidoux A, Shibata H, Terz J, Paterson AH, Feldman MI, Farrar W, Evans J and Lickley HL (1996) Five versus more than five years of tamoxifen therapy for breast cancer patients with negative lymph nodes and estrogen receptor-positive tumors. *J Natl Cancer Inst* **88**: 1529–1542

Fisher B, Dignam J, DeCillis DL, et al (1997) The worth of chemotherapy and tamoxifen (TAM) over TAM alone in node-negative patients with estrogen-receptor positive invasive breast cancer: first results from NSABP B-20 (abstract). *Proc Am Soc Clin Oncol* **16**, 1a

Forbes JF (1997) The control of breast cancer: the role of tamoxifen. *Semin Oncol* **24** (suppl. 1): (S1) 5–19

Jaiyesimi IA, Buzdar AU, Decker DA and Hortobagyi GN (1995) Use of tamoxifen for breast cancer: twenty-eight years later [see comments]. *J Clin Oncol* **13**: 513–529

Jonat W, Kaufmann M, Blamey RW, Howell A, Collins JP, Coates A, Eiermann W, J'anicke F, Njordenskold B and Forbes JF (1995) A randomised study to compare the effect of the luteinising hormone releasing hormone (LHRH) analogue goserelin with or without tamoxifen in pre- and perimenopausal patients with advanced breast cancer. *Eur J Cancer* **31A**: 137–142

Katzenellenbogen BS, Montano MM, Ekena K, Herman ME and McInerney EM (1997) William L. Maguire Memorial Lecture. Antiestrogens: mechanisms of action and resistance in breast cancer. *Breast Cancer Res Treat* **44**: 23–38

Koss LG, Schreiber K, Oberlander SG, Moussouris HF and Lesser M (1984) Detection of endometrial carcinoma and hyperplasia in asymptomatic women. *Obstet Gynecol* **64**: 1–11

Love RR, Mazess RB, Barden HS, et al (1992) Effects of tamoxifen on bone mineral density in postmenopausal women with breast cancer. *N Engl J Med* **326**: 852–856

Love RR, Wiebe DA, Feyzi JM, Newcomb PA and Chappell RJ (1994*a*) Effects of tamoxifen on cardiovascular risk factors in postmenopausal women after 5 years of treatment. *J Natl Cancer Inst* **86**: 1534–1539

Love RR, Wiebe DA, Feyzi JM, et al (1994*b*) Effects of tamoxifen on cardiovascular risk factors in postmenopausal women after 5 years of treatment. *J Natl Cancer Inst* **86**: 1534–1539

MacMahon B (1997) Overview of studies on endometrial cancer and other types of cancer in humans: perspectives of an epidemiologist. *Semin Oncol* **24** (suppl. 1): (S1) 122–139

Muss HB (1992) Endocrine therapy for advanced breast cancer: a review. *Breast Cancer Res Treat* **21**: 15–26

Nayfield SG and Gorin MB (1996) Tamoxifen-associated eye disease: a review. *J Clin Oncol* **14**: 1018–1026

Nolvadex Adjuvant Trial Organisation (1983) Controlled trial of tamoxifen as adjuvant agent in management of early breast cancer. *Lancet* **i**: 257–261

Peto R (1996) Five years of tamoxifen – or more? *J Natl Cancer Inst* **88**: 1791–1793

Powles TJ (1997) Efficacy of tamoxifen as treatment of breast cancer. *Semin Oncol* **24**(suppl. 1): S48–54

Powles TJ, Hickish T, Kanis JA, Tidy A and Ashley S (1996) Effect of tamoxifen on bone mineral density measured by dual-energy X-ray absorptiometry in healthy premenopausal and postmenopausal women. *J Clin Oncol* **14**: 78–84

Quinn M and Allen E (1995) Changes in incidence of and mortality from breast cancer in England and Wales since introduction of screening. *Br Med J* **311**: 1391–1395

Rose C and Mouridsen HT (1988) Endocrine therapy of advanced breast cancer. *Acta Oncol* **27**: 721–728

Swedish Breast Cancer Cooperative Group (1996) Randomized trial of two versus five years of adjuvant tamoxifen for postmenopausal early stage breast cancer. *J Natl Cancer Inst* **88**: 1543–1549

Tormey DC, Gray R and Falkson HC (1996) Postchemotherapy adjuvant tamoxifen therapy beyond five years in patients with lymph node-positive breast cancer. *J Natl Cancer Inst* **88**: 1828–1833

© Cancer Research Campaign 1998